# B Cell Responses to CpG Correlate with CXCL16 Expression Levels in Common Variable Immunodeficiency

**DOI:** 10.1100/2012/960219

**Published:** 2012-02-01

**Authors:** Vassilios Lougaris, Manuela Baronio, Massimiliano Vitali, Giacomo Tampella, Annarosa Soresina, Raffaele Badolato, Alessandro Plebani

**Affiliations:** Pediatrics Clinic and Institute of Molecular Medicine “A. Nocivelli”, University of Brescia, piazzale Spedali Civili 1, 25123 Brescia, Italy

## Abstract

Broad Toll-like receptor 9 (TLR9) signalling defects after CpG *in vitro* stimulation have been described in common variable immunodeficiency (CVID). CXCL16, a surface receptor, was recently shown to influence cell responses to CpG. We evaluated the expression and function of CXCL16 on B cells from healthy controls and CVID patients. We report that CXCL16 is normally expressed on B cells throughout peripheral maturation. Decreased B cell expression of CXCL16 was observed in a subgroup of CVID patients that correlated with defective *in vitro* responses to CpG (such as upregulation of CD69, CD86, AICDA, IL-6, and TLR9). Our data suggest that expression levels of a surface receptor, namely, CXCL16, correlate with B cell responses mediated by TLR9 in common variable immunodeficiency.

## 1. Introduction

Common variable immunodeficiency (CVID) is the most common symptomatic primary immunodeficiency [1–4]. Defective B cell maturation in memory B cells and plasma cells is largely accredited to represent the main pathogenetic mechanism resulting in hypogammaglobulinemia and consequently recurrent infections in the affected patients. Clinical manifestations may also include autoimmune phenomena, splenomegaly and lymphomas [2, 3, 5]. Genetic causes are only known in 10–12% of patients, underlying the important heterogeneity and complexity of this disorder. Disease-causing mutations in ICOS, CD19, CD81, and disease-predisposing mutations in TACI, BAFF-R, and CD20 have been reported in a minority of CVID patients [6–12]. Of note, with the exception of ICOS, all of these affect B cell specific genes, in keeping with the notion that impaired B cell homeostasis is a hallmark of CVID.

In recent years, the importance of components of the innate immune system, namely, Toll-like receptor family members (TLRs), was underlined and a direct link between innate and adaptive immunity was established. TLR9 in particular, selectively expressed in the cytoplasm of B cells, promotes B cell activation and maturation in response to CpG (oligodeoxynucleotides: oligonucleotides containing selected CpG-DNA sequences) stimulation *in vitro *[13]. *In vitro* studies showed that CVID patients presented profound defects in response to *in vitro* CpG stimulation. In particular, CVID B cells presented defective upregulation of CD86 after CpG stimulation, failed to increment IL-6 and IL-10 production, and failed to upregulate activation-induced cytosine deaminase (AICDA) [14, 15]. However, the molecular mechanisms underlying impaired response to CpG in patients with CVID remain undefined.

Recent studies have suggested that the interaction between CXCL16 and CXCR6 might influence the inflammatory responses and the magnitude of the immune response to CpG by specifically influencing the cellular uptake, subcellular localization, and cytokine profile [16].

 So far, CXCL16 has been reported to be present in a membrane bound and a soluble form and appears to have multiple roles: it functions as an endocytic receptor, as an adhesion molecule, and as a chemokine which binds the orphan G-protein receptor CXCR6 [17]. Several functions have been attributed to membrane-bound CXCL16. Through its chemokine domain, it promotes adhesion and phagocytosis of both Gram-positive and Gram-negative bacteria, as well as chemotaxis of T and NKT cells [18]. CXCL16 is expressed by different cell types such as monocytes, pDCs, smooth muscle cells, while the expression on B cells is not well characterized yet [19]. The receptor CXCR6 on the other hand is expressed by different T cell subsets, namely, CD8 T cells and CD4 effector memory and central memory T cells, but not naïve CD4 T cells [20]. In light of the role of CXCL16 in modulating cellular responses to CpG, and of the demonstration that B lymphocytes from CVID patients have an impaired response to CpG, we have assessed the expression of CXCL16 on the surface of B lymphocytes from CVID patients and correlated such expression with CVID B cell responses to CpG.

The molecular basis of defective B cell responses to CpG in CVID remains unknown. We hypothesized that *in vitro* B cell responses to CpG stimulation in CVID may be dependent on CXCL16. Our data provide evidence that CVID B cell responses to CpG correlate with CXCL16 expression levels.

## 2. Materials and Methods

### 2.1. Patients

Fifteen patients affected with CVID were included in this study; male to female ratio was close to 1 : 1 (8 male versus 7 female). Summary of patients' clinical and immunological characteristics is shown in Table 1. Diagnosis of CVID was made according to the ESID criteria (http://www.esid.org/). CVID patients are under regular followup at the Outpatient Clinic of the Pediatric Immunology and Rheumatology Unit, Children's Hospital, Brescia, Italy. All patients were under regular immunoglobulin replacement therapy. This study was conducted in accordance with the guidelines of the World Medical Association's Declaration of Helsinki (most recent revision). This study was reviewed and approved from the Hospital's Ethical Committee. Informed consent was obtained from all patients and healthy controls (matched for age and sex). 

### 2.2. Primers, PCR, and Genetic Sequencing

Primers were designed including the flanking regions of exons 1 to 5 of the *CXCL16* encoding gene (available upon request). PCR products were then purified, and direct gene sequencing of all exons of the *CXCL16* gene was performed using an ABI PRISM 310 sequencer. Sequences were analyzed using the Sequence Navigator software (SeqScape v2.6).

### 2.3. Cell Separation, Cell Stimulation, Flow Cytometry, and Real Time PCR

PBMCs from healthy controls and CVID patients were separated from peripheral whole blood samples using Ficoll (Lympholyte H sterile liquid, Cedarlane Diagnostics). Tonsillar B cells were isolated from healthy tonsils after tissue digestion, Ficoll separation, and B cell separation (Easy Sep Human Enrichment Kit, Stem Cell). Cell separation efficiency was verified by flow cytometry with B cell purity >90%. Cells were cultured in 96-well plates (200.000 cells per well) in RPMI added with 10% FCS (EuroClone), Penicillin-Streptomycin (EuroClone), and L-Glutamine (EuroClone) in the presence or absence of CpG ODN 2216 (Labogen) at a final concentration of 0.5 *μ*M. Cells were harvested at time 0 and after 24-hour incubation with or without CpG, washed with PBS 1% FCS, and then marked with specific antibodies (CD19-PerCP, CD20-PerCP, CD27-PE, IgD-FITC, CD69-FITC, CD86-PE)(BD, Becton Dickinson), incubated for 20 minutes at 4°C, washed twice, and then acquired with a FACS-Calibur Cytometer (BD). CXCL16 expression was evaluated using a biotinylated CXCL16 monoclonal antibody (incubated for 20 minutes at 4°C, washed twice) followed by a secondary staining with streptavidin-PE or streptavidin-APC (incubated for 20 minutes at 4°C, washed twice). Flow cytometric analysis was performed using the Flowjo software. In alternative, after 24 hours of incubation with or without CpG, total RNA was extracted and Real-Time PCR was performed for Tlr-9, Aicda, *Il-6* genes, according to the manufacturer's protocol (Applied Biosystems). For the TNF-*α* blockade experiments, cells were incubated with monoclonal anti-TNF-*α* for 48 hours at 50 ug/mL and then CpG was added as above. Real Time PCR was analyzed with the ABI PRISM 7000 SDS software.

### 2.4. Statistical Analysis

The *χ* ^2^ test was used for the analysis of the allelic frequencies, for the CXCL16 expression levels and Real-Time experiments between CVID patients and healthy controls. The findings were considered significant when the *P* value was lower than 0.001 (*P* < 0.001).

## 3. Results

### 3.1. CXCL16 Expression on Primary B Cells

**Figure 1:**
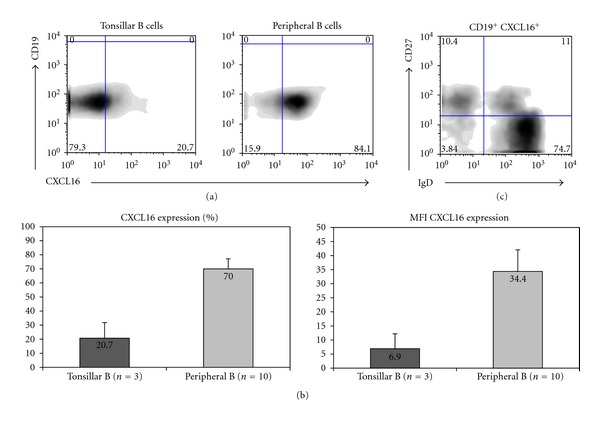
CXCL16 expression on primary B cells. (a) CXCL16 expression on tonsillar CD19^+^ B cells and peripheral CD19^+^B cell subsets from healthy control (representative experiment). (b) Histogram-depicting percentage and mean fluorescence intensity of CXCL16^+^ CD19^+^ tonsillar B cells and peripheral B cells from healthy donors (mean of three different experiments for tonsillar B cells and ten healthy controls for peripheral B cells). (c) CXCL16 expression in the different maturative stages of peripheral B cells (representative of three different experiments).

CXCL16 expression has been described in various cell types such as dendritic cells, macrophages, podocytes, and others; however, its expression on B cells has not been clearly established. We show that human B cells from peripheral blood and tonsils express CXCL16 (Figures 1(a) and 1(b)). Such expression appears higher in the periphery (Figure 1(a): left panel) when compared to tonsillar B cells (Figure 1(a): right panel). Expression of CXCL16 on B cells does not appear to depend on the maturational stage, as naïve CD21^hi^ CD27^−^IgD^+^, nonswitched CD21^hi^ CD27^+^IgD^+^, and switched CD21^hi^ CD27^+^IgD^−^ B cells express this protein (Figure 1(c)).

### 3.2. CXCL16 Expression Is Reduced on CVID B Cells

**Figure 2:**
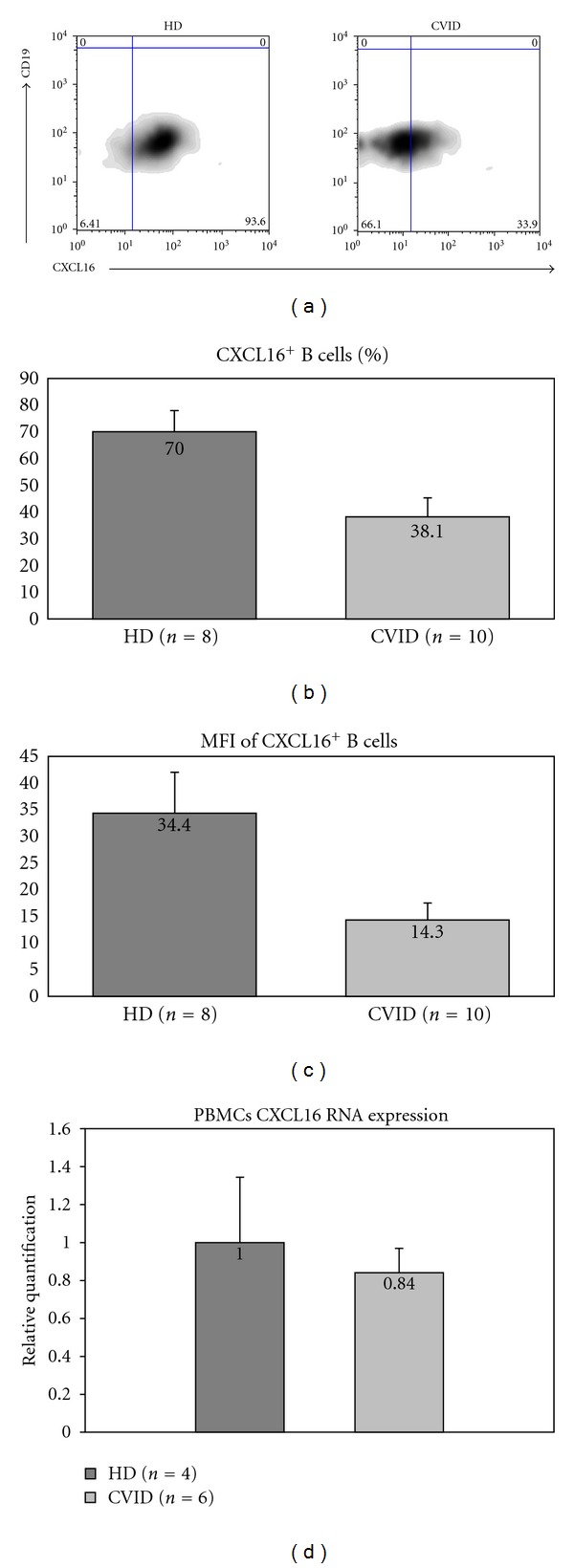
CXCL16 expression in CVID. (a) Reduced expression of CXCL16 on CVID B cells (representative experiment). (b) Mean of CXCL16^+^ B cells in healthy controls (number = 8) and CVID patients (number = 10). (c) Mean fluorescence intensity of CXCL16 on CD19^+^ B cells in cells in healthy controls (number = 10) and CVID patients (number = 10) (values expressed in mean). Graph bars show standard error (SE). (d) mRNA levels of CXCL16 from healthy controls and CVID patients.

We then evaluated the expression of CXCL16 on CVID B cells.  Figure 2 shows a representative experiment of B cell CXCL16 expression confronting a healthy control (HD) and a CVID patient: the expression of CXCL16 is evidently reduced in the CVID patient (Figure 2(a)).  Figure 2(b) shows the mean of 10 healthy controls and 10 CVID patients. The percentage of peripheral B cells from CVID patients expressing CXCL16 was significantly lower than in age-matched healthy controls (70% versus 38.1%, *P* = 0.0277) (Figure 2(b)). Furthermore, the mean fluorescence intensity of CXCL16 expression at the cell surface was also reduced in CVID patients versus controls (MFI: 14.3 versus 34.4, resp., *P* < 0.001) (Figure 2(c)). Direct gene sequencing of the *CXCL16* gene was performed in order to evaluate whether the reduced surface expression of the CXCL16 protein was due to genetic alterations. Seven already reported SNPs (rs2250333, rs1051009, rs1050997, rs2277680, rs2304970, rs10509989, and rs1876444) were found in different combinations among the CVID patients. The allelic frequencies of these variants in CVID patients were similar to those observed in healthy controls (data not shown). Furthermore, CXCL16 mRNA expression levels were similar between CVID patients and healthy controls (Figure 2(d)).

### 3.3. CXCL16 and CpG

**Figure 3:**
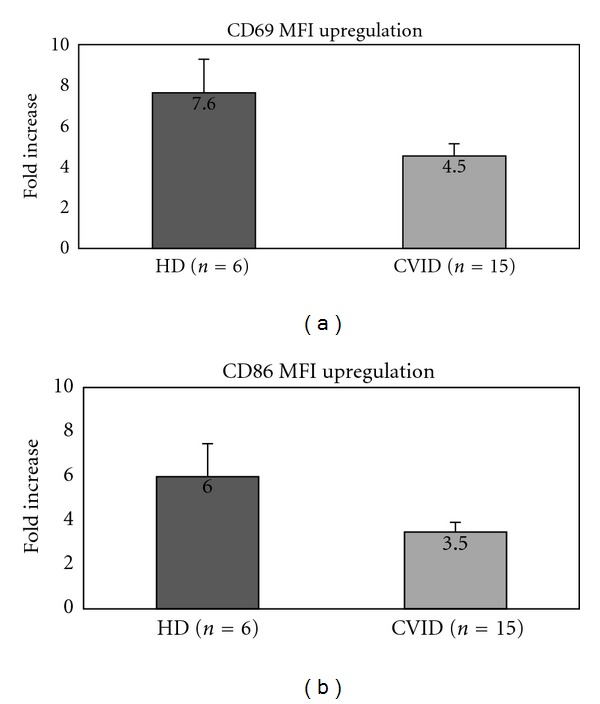
Defective upregulation of activation markers (CD69, CD86) after *in vitro* stimulation with CpG in CVID. (a) Fold increase in CD69 expression in healthy controls and CVID patients CVID (mean of six and fifteen samples, resp.). (b) Fold increase in CD86 expression in healthy controls and CVID patients (mean of six and fifteen samples, resp.). Graph bars show standard error (SE).

Since CXCL16 expression has been reported to influence immune cell responses after CpG stimulation [16], we decided to investigate whether the reduced CXCL16 expression on CVID B cells correlated with specific biological responses *in vitro*. To address this issue, PBMCs were incubated with CpG and tested for upregulation of the activation markers CD69 and CD86 after 24 hours of incubation (Figure 3(a)). CVID B cells presented a significant defect in CD69 upregulation after CpG *in vitro* stimulation: the mean fold increase in MFI was 59.4% lower than in healthy controls (4.54 versus 7.64, resp.). Similar findings were observed when the upregulation of CD86 after *in vitro* CpG stimulation was tested (Figure 3(b)): the mean fold increase in MFI was 58.7% lower than in healthy controls (3.47 versus 5.97, resp.).

**Figure 4:**
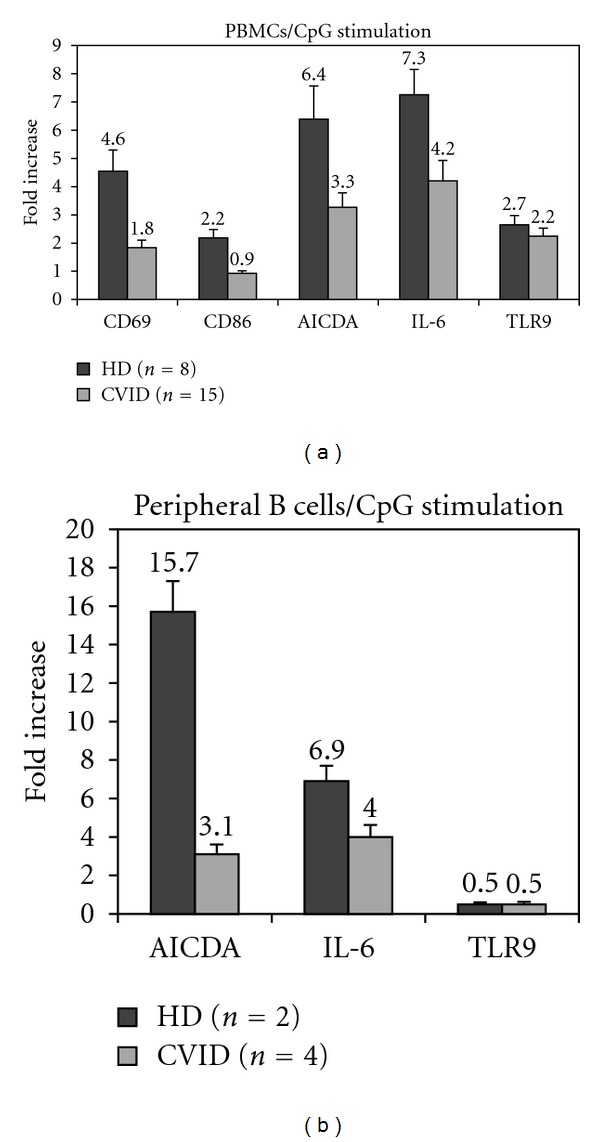
Defective upregulation target genes (mRNA levels) after *in vitro* stimulation with CpG in CVID. (a) Fold increase of CD69, CD86, TLR9, AICDA, and IL-6 at the mRNA level after CpG stimulation. Experiments were performed with PBMCs in triplicates for all patients (*n* = 15) and healthy controls (*n* = 8). (b) Fold increase of TLR9, AICDA, and IL-6 at the mRNA level after CpG stimulation. Experiments were performed with purified B cells from patients (*n* = 4) and healthy controls (*n* = 2). Graph bars show standard error (SE).

B cell responses to CpG may also be measured by mRNA upregulation of specific target genes such as *TLR9*, *AICDA*, *IL-6*, *CD69*, and *CD86*. We therefore performed Real-Time PCR for the above mentioned genes after CpG *in vitro* stimulation for 24 hours (Figure 4(a)). TLR9 upregulation was similar between healthy controls and CVID patients (*P* = 0.7073-n.s). However, we observed a statistically significant difference in the upregulation of CD69, CD86, AICDA, and IL-6; in particular, CVID B cells fail to up-regulate CD69, CD86, AICDA, and IL-6 mRNA after CpG stimulation when compared to healthy controls (61%, 59%, 49%, and 43% reduced upregulation, resp.). These results were convalidated by performing the same experiment with purified peripheral B cells from healthy controls and CVID patients with similar results (Figure 4(b)). 

### 3.4. CXCL16^high^ B Cells in CVID

**Figure 5:**
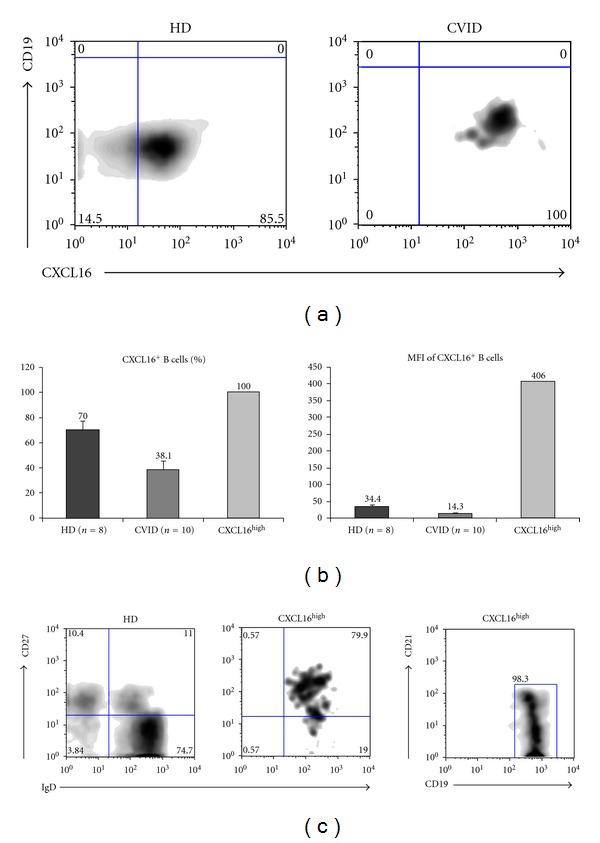
Identification of CXCL16^high^ B cells. (a) Elevated expression of CXCL16 on CVID B cells when compared to healthy control. (b) Elevated expression of CXCL16 on CVID B cells when compared to the mean expression levels of CVID patients and healthy controls both as percentage (left panel) and mean fluorescence intensity (right panel). (c) Characterization of maturational status of CXCL16^high^ B cells by expression of CD27, IgD, and CD19, CD21. Graph bars show standard error (SE).

During our evaluation of CXCL16 expression, we identified the presence of CXCL16^high^ expressing B cells in CVID.  Figure 5(a) shows the elevated expression of CXCL16 on the peripheral B cells from the CVID patient (Patient 10; see Table 1) when compared to the healthy control. We then compared the expression of CXCL16 between the CXCL16^high^ CVID patient, eight healthy controls and ten CVID patients (Figure 5(b)). The totality of the CXCL16^high^ CVID B cells express CXCL16, while such percentage is around 70% for healthy controls and is further reduced in CVID patients (40%) (Figure 5(b) left panel). Interestingly, the mean fluorescence intensity is particularly elevated in this patient: more than 10-fold when compared to healthy controls and almost 29-fold when compared to the CVID patients (420 versus 40 for the healthy controls and 15 for the CVID patients) (Figure 5(b) right panel).

Furthermore, we tried to better characterize the maturational state of the CXCL16^high^ B cells by multicolor flow-cytometry (Figure 5(c)). We found that these cells present defective peripheral maturation as they only present the CD27^+^IgD^+^ population. Interestingly, the totality of CXCL16^high^ B cells is CD19^hi^CD21^low^ (Figure 5(c) right panel) confirming the presence of this B cell subset in patients with autoimmune phenomena (CVID patient affected with autoimmune enteropathy).

### 3.5. CXCL16^high^ Expression on CVID B Cells Evokes a Vigorous Response to CpG

**Figure 6:**
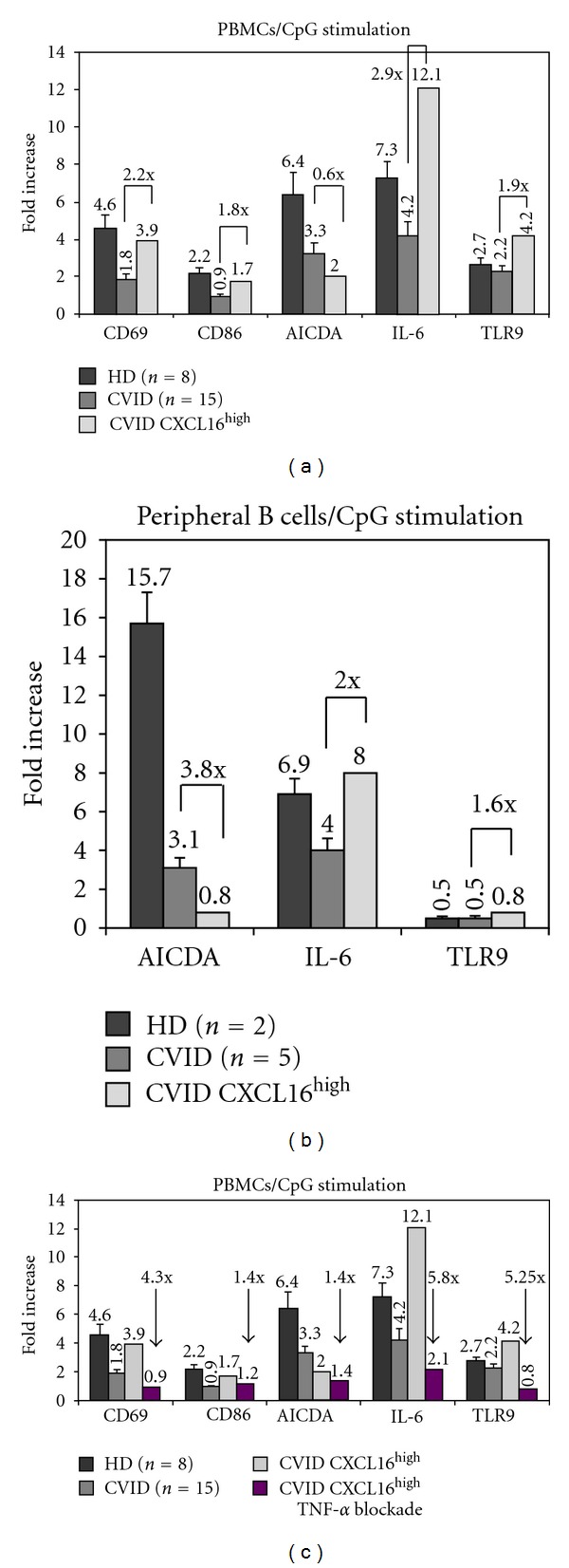
CXCL16^high^ B cell responses to CpG. (a) Evaluation of target gene (mRNA levels) upregulation (mRNA levels) after *in vitro* stimulation with CpG. Fold increase of CD69, CD86, TLR9, AICDA, and IL-6 at the mRNA level after CpG stimulation. Experiments were performed with PBMCs in triplicates for all patients (*n* = 15) and healthy controls (*n* = 8). (b) Fold increase of TLR9, AICDA, and IL-6 at the mRNA level after CpG stimulation. Experiments were performed with purified B cells from patients (*n* = 5) and healthy controls (*n* = 2). Graph bars show standard error (SE). (c) Evaluation of target gene (mRNA levels) upregulation (mRNA levels) after TNF-*α* blockade and *in vitro* stimulation with CpG. Fold variation of CD69, CD86, TLR9, AICDA, and IL-6 at the mRNA level is showed. Experiments were performed with PBMCs in triplicates for all patients (*n* = 15) and healthy controls (*n* = 8). Graph bars show standard error (SE).

The identification of the CXCL16^high^ B cell population in CVID led us to investigate their *in vitro* responses to CpG stimulation and compare them with those from healthy controls and CVID patients with low CXCL16 expression (Figures 6(a) and 6(b)). 

CXCL16^high^ CVID B cells presented a higher upregulation of CD69 and CD86 when compared to CVID patients with low CXCL16 expression (2.2 and 1.8 fold, resp.); interestingly, such upregulation was similar to that observed in healthy controls (Figure 6(a)).

Surprisingly, CXCL16^high^ B cells showed a vigorous response to CpG stimulation as defined by upregulation of IL-6 and TLR9 mRNA when compared to CVID patients with low CXCL16 expression (2.9 and 1.9 fold, resp.) and to healthy controls (1.65 and 1.55 fold, resp.) (Figure 6(a)). AICDA mRNA levels were lower in CXCL16^high^ B cells after CpG stimulation. These results were convalidated by performing the same experiment with purified peripheral B cells from healthy controls and CVID patients with similar results (Figure 6(b)). Inflammatory and autoimmune phaenomena are frequently treated with monoclonal blocking antibodies to TNF-*α* (Infliximab) in CVID (21–24). Considering the clinical data (CVID patient with enteropathy), we tested whether the vigorous responses of the CXCL16^high^ B cells were dependent on TNF-*α*. TNF-*α* blockade abrogated B cell responses to CpG. More in detail, we noticed a 4.3 fold reduction in the up-regulation of CD69 mRNA, and more than 5-fold reduction in the up-regulation of IL-6 and TLR9 (5.8 and 5.25 fold, resp.). Reduction in CD86 and AICDA up-regulation was less significant, but still present (1.4 fold each) (Figure 6(c)).

## 4. Discussion

Common variable immunodeficiency (CVID) is a complex disorder where little is known on the biological mechanisms underlying B cell defective responses. Genetic studies in the last years have shed light in the genetic causes of a limited number of CVID patients (10–12%) [6–12]. The genetic defects so far identified are B cell specific with the exception of ICOS deficiency. On the other hand, components of the innate immunity, in particular the family of Toll-like receptors (TLRs), were recently linked to the homeostasis of the adaptive arm of the immune system. TLR9 signalling in particular has been shown to be important for B cell activation and maturation [13]. Defects in the TLR9 signalling pathway after CpG stimulation were recently reported in CVID patients [14, 15] confirming such link. CXCL16, a membrane bound scavenger receptor, has been shown to influence responses to CpG [16]. CXCL16 expression has been described on macrophages, dendritic cells, podocytes, epithelial cells, tumor cells; however, the expression of CXCL16 on B cells is not well established yet. We show that CXCL16 is normally expressed on primary B cells, both from healthy tonsils and peripheral blood. We describe the reduced expression of CXCL16, both as percentage and mean fluorescence intensity, on a group of CVID patients' B cells to be associated with defective *in vitro* responses to CpG. Direct gene sequencing of the *CXCL16* encoding gene evidenced the presence of seven already reported SNPs at similar allelic frequencies as found in healthy controls; such mutations did not affect the mRNA levels. We also show that CXCL16 is normally expressed during all stages of peripheral B cell maturation, suggesting that the reduced expression in CVID is not related to the altered peripheral B cell differentiation, a hallmark of CVID. It is well known that in CVID, T and B cell interactions may be altered causing defective/altered germinal center reaction, thus resulting in defective generation of memory B cells and plasma cells [1, 3–5]. Since CXCL16 is a strong chemoattractant for CXCR6 expressing T cells, it could be hypothesized that reduced expression of CXCL16 on CVID B cells may influence in a negative manner the crosstalk between T and B cells in CVID. Further studies will be needed to address this issue. In addition, CXCL16 has been shown not only to recruit activated T lymphocytes but also to play a direct role in the binding and phagocytosis of bacteria (both Gram-positive and Gram-negative ones) by professional antigen-presenting cells [21, 22]. It can be therefore proposed that low CXCL16 expression on CVID B cells may contribute to noneffective clearance from bacterial infections, another hallmark of CVID.

The correlation between B cell CXCL16 expression levels and B cell responses to CpG was further underlined by the identification of CVID B cells with high expression levels of CXCL16 (CXCL16^high^). These CXCL16^high^ B cells express the protein with a mean fluorescence intensity almost 30-fold superior to that observed in other CVID patients. CXCL16^high^ B cells appear to be associated with autoimmune phenomena in CVID [23–26], autoimmune enteropathy in our case. Interestingly, CXCL16^high^ B cells are exclusively CD19^hi^CD21^low^, confirming the role of this B cell subpopulation in autoimmune manifestations. CXCL16^high^ B cells showed a vigorous response to CpG stimulation *in vitro*, for some aspects even superior to healthy controls (IL-6 and TLR9 upregulation). Moreover CXCL16^high^ B cells are associated with autoimmune and inflammatory phaenomena and show a vigorous response to CpG in a TNF-*α* dependent manner. It appears therefore plausible that CXCL16 may become an additional marker for B cells in CVID patients with autoimmune and inflammatory phaenomena, together with CD21^low^.

In conclusion, we report that the expression levels of CXCL16 on B cells can predict the extent of B cell responses to CpG in CVID patients. The reduced expression of CXCL16 on B cells correlates with defective cell activation; on the other hand, elevated expression levels of CXCL16 on B cells (CXCL16^high^) correlates with a vigorous response to CpG and appear to be associated with autoimmune and inflammatory phaenomena, at least in our case. It is evident that a larger number of CVID patients with autoimmune phaenomena needs to be tested to further confirm this finding.

To our knowledge, this constitutes the first report which links the expression levels of a surface receptor with the cell responses mediated by an intracellular TLR and is associated with a disease in humans, namely, common variable immunodeficiency.

## Figures and Tables

**Table 1:** Summary of the clinical and immunological features of CVID patients included in this study (age of onset, immunoglobulin serum levels at diagnosis, percentage of CD19 positive B cells, and presence/absence of autoimmune manifestations and splenomegaly).

	Age of onset (years)	IgG (mg/dL)	IgA (mg/dL)	IgM (mg/dL)	CD19 (%)	Autoimmunity	Splenomegaly
Patient 1	3	274	22	25	18	n	n
Patient 2	1	160	4	22	24	n	y
Patient 3	40	313	2	37	15	n	y
Patient 4	1	239	46	18	24	n	n
Patient 5	7	408	4	17	12	n	n
Patient 6	6	62	367	180	18	n	y
Patient 7	3	69	6	14	12	n	y
Patient 8	2	330	26	10	25	n	y
Patient 9	13	495	9	7	2	n	n
Patient 10	7	160	14	14	3	y	n
Patient 11	9	432	184	24	2	n	y
Patient 12	21	463	2	263	11	n	n
Patient 13	34	59	1	20	10	n	n
Patient 14	1	291	5	6	20	n	y
Patient 15	24	85	4	16	2	y	y

Median	7	256	6	18	12		
